# Systematic studies of the interactions between a model polyphenol compound and microbial β-glucosidases

**DOI:** 10.1371/journal.pone.0181629

**Published:** 2017-07-20

**Authors:** Viviam M. da Silva, Juliana A. P. Sato, Juscemácia N. Araujo, Fabio M. Squina, João R. C. Muniz, Karin A. Riske, Wanius Garcia

**Affiliations:** 1 Centro de Ciências Naturais e Humanas, Universidade Federal do ABC (UFABC), Santo André, São Paulo, Brazil; 2 Programa de Processos Tecnológicos e Ambientais, Universidade de Sorocaba (UNISO), Sorocaba, São Paulo, Brazil; 3 Instituto de Física de São Carlos (IFSC), Universidade de São Paulo (USP), São Carlos, São Paulo, Brazil; 4 Departamento de Biofísica, Universidade Federal de São Paulo, São Paulo, Brazil; Islamic Azad University Mashhad Branch, ISLAMIC REPUBLIC OF IRAN

## Abstract

Lignin is a major obstacle for cost-effective conversion of cellulose into fermentable sugars. Non-productive adsorption onto insoluble lignin fragments and interactions with soluble phenols are important inhibition mechanisms of cellulases, including β-glucosidases. Here, we examined the inhibitory effect of tannic acid (TAN), a model polyphenolic compound, on β-glucosidases from the bacterium *Thermotoga petrophila* (TpBGL1 and TpBGL3) and archaeon *Pyrococcus furiosus* (PfBGL1). The results revealed that the inhibition effects on β-glucosidases were TAN concentration-dependent. TpBGL1 and TpBGL3 were more tolerant to the presence of TAN when compared with PfBGL1, while TpBGL1 was less inhibited when compared with TpBGL3. In an attempt to better understand the inhibitory effect, the interaction between TAN and β-glucosidases were analyzed by isothermal titration calorimetry (ITC). Furthermore, the exposed hydrophobic surface areas in β-glucosidases were analyzed using a fluorescent probe and compared with the results of inhibition and ITC. The binding constants determined by ITC for the interactions between TAN and β-glucosidases presented the same order of magnitude. However, the number of binding sites and exposed hydrophobic surface areas varied for the β-glucosidases studied. The binding between TAN and β-glucosidases were driven by enthalpic effects and with an unfavorable negative change in entropy upon binding. Furthermore, the data suggest that there is a high correlation between exposed hydrophobic surface areas and the number of binding sites on the inhibition of microbial β-glucosidases by TAN. These studies can be useful for biotechnological applications.

## Introduction

Lignocellulosic biomass is composed mainly of cellulose, hemicellulose and lignin, and the proportion of each constituent varies from plant to plant (for example between softwoods, hardwoods, or grasses) [1–3]. Cellulose represents the largest carbohydrate fraction of biomass and it is used in several biotechnological processes, especially in the field of second-generation bioethanol production [2]. Many different pretreatment methods can be employed to remove hemicellulose and lignin to improve the efficiency of the enzymatic hydrolysis of cellulose [4–6]. Efficient enzymatic hydrolysis of cellulose to glucose requires hydrolytic cellulases working synergistically: endoglucanases (E.C. 3.2.1.4), cellobiohydrolases (E.C. 3.2.1.91), and β-glucosidases (E.C. 3.2.1.91) [3,7]. Endoglucanases randomly cleave the cellulose and create new chain ends, while cellobiohydrolases cleave off cellobiose from the end of cellulose chains [7,8]. The β-glucosidases hydrolyze the released cellobiose and other soluble oligosaccharides into glucose [9]. Additionally, AA9 enzymes which are copper-dependent lytic polysaccharide monooxygenases, together with cellobiose dehydrogenase, can improve cellulose break down via oxidative mechanisms [10]. Cellobiose is a potent inhibitor of both endoglucanases and cellobiohydrolases, consequently, the β-glucosidases are essential enzymes preventing cellobiose accumulation and maintaining the hydrolysis rates of cellulose over the time [11].

Lignin is an aromatic complex polymer and a major obstacle for cost-effective conversion of cellulose into fermentable sugars [3]. Lignin composition may vary from plant to plant, and is composed mainly of phenylpropane units (guaiacyl propanol, syringyl propanol, and *p*-hydroxyphenyl propanol) [12,13]. The enzymatic hydrolysis of cellulose to glucose by cellulases may be inhibited in the presence of residual lignin produced as a result of pretreatment, the latter which frequently results in considerable amounts of insoluble lignin fragments and soluble phenolics [14–19]. The mechanism of enzyme inhibition may involve both non-productive adsorption of cellulases onto insoluble lignin fragments, as well as interactions with solubilized phenolics [14–19]. Therefore, a better understanding of the interaction of cellulases with lignin and its soluble derivatives can help to improve the development of biofuel production technologies.

Polyphenols are well-known to have high affinity to bind proteins and other biomolecules [20–24]. The interaction between phenolics and proteins may be influenced by several parameters as temperature, pH, ionic strength, protein folds and types of phenolics [24]. In the present study, we examined the interaction and the inhibitory effect of tannic acid (TAN) on β-glucosidases from the bacterium *Thermotoga petrophila* (belonging to the families GH1 and GH3) and archaeon *Pyrococcus furiosus* (belonging to the family GH1) [25–26]. As previously described, TAN can be used as a realistic polyphenol model compound for lignin and soluble phenolics [18]. Soluble phenolics produced by pretreatments processes can inhibit and inactivate β-glucosidases under industrial applications [3,15,16]. Our results showed that the inhibition effects were found to be TAN concentration-dependent. In an attempt to better understand the inhibitory effect, we used high sensitivity isothermal titration calorimetry (ITC) to investigate the thermodynamic binding properties of the interaction between TAN and β-glucosidases. The exposed hydrophobic surface areas in β-glucosidases were also analyzed using the fluorescent probe 1-anilinonaphthalene-8-sulfonate (ANS) and compared with the results of inhibition and ITC.

## Materials and methods

### Materials

All chemicals and reagents used in this study were of the highest purity grade. LB medium, kanamycin sulfate, isopropyl-β-D-thiogalactopyranoside (IPTG), nickel-nitrilotriacetic acid resin (Ni-NTA), imidazole, 4-nitrophenyl-β-D-glucopyranoside (pNPG), 1-anilinonaphthalene-8-sulfonate (ANS), tannic acid (TAN, molecular weight of 1,701.2 g/mol) and Triton X-100 (approximate molecular weight of 625 g/mol) were purchased from Sigma–Aldrich.

### Expression and purification of recombinant β-glucosidases

The expression in *Escherichia coli* BL21(DE3) and purification of recombinant β-glucosidases from bacterium *Thermotoga petrophila* (TpBGL1 and TpBGL3) and archaeon *Pyrococcus furiosus* (PfBGL1) were carried out as described previously [25–26]. The purification of recombinant β-glucosidases were performed using affinity and size exclusion chromatographies. The purity of the final β-glucosidases were verified by 15% SDS-PAGE. The concentrations of the recombinant β-glucosidases were determined by UV absorbance at 280 nm using theoretical extinction coefficients based on the amino acid sequence. The extinction coefficients (ε) were calculated for the monomers using the ProtParam tool [27]. The theoretical coefficients employed were ε_280nm_ = 121,240 M^−1^cm^−1^ for TpBGL1, ε_280nm_ = 102,930 M^−1^cm^−1^ for TpBGL3 and ε_280nm_ = 131,210 M^−1^cm^−1^ for PfBGL1. The purified β-glucosidases were stored at 4°C and used until one week after the purification. The expected molecular mass (monomer) for TpBGL1, TpBGL3 and PfBGL1 are 52, 83 and 55 kDa, respectively.

### Inhibition assays

The standard enzymatic assay for β-glucosidase activity was performed according to previously described method and using 4-nitrophenyl-β-D-glucopyranoside (pNPG) as substrate [25,28]. Each reaction was composed of 100 μL of 250 mM acetate-borate-phosphate buffer solution adjusted at different pH values (pH 6 for TpBGL1, pH 5 for PfGH1 and pH 4 for TpBGL3) containing 1 μM β-glucosidase, 1 mM pNPG and different TAN concentrations (from 0 to 0.5 mM). In each case, before adding the pNPG the enzyme was incubated during 10 min with TAN. After, each reaction was incubated at 70°C during 3 min. Subsequently, the reaction was stopped by addition of 100 μL of 1 M Na_2_CO_3_ and the releasing of p-nitrophenol was monitored colorimetrically at 405 nm using a microplate reader (Thermo Fisher Scientific). All β-glucosidase assays presented in this study were done in triplicate.

### Fluorescence experiments (ANS assay)

Fluorescence emission measurements were performed on a steady-state spectrofluorimeter Varian (model Cary Eclypse) equipped with a refrigerated circulator. Fluorescent dye binding experiments with 1-anilino-8-naphthalenesulfonic acid (ANS) were performed in order to probe hydrophobic regions in the three microbial β-glucosidases used in this study [29,30]. A fixed concentration of β-glucosidase (10 μM) in 250 mM acetate-borate-phosphate buffer solution adjusted at different pH values (pH 6 for TpBGL1, pH 5 for PfGH1 and pH 4 for TpBGL3) was mixed at different ANS concentrations (from 0 to 0.12 mM). The excitation wavelength was set at 360 nm at 25°C. Fluorescence emission spectra were recorded from 400 to 650 nm (monitored at 490 nm) from samples loaded into a 1 cm path-length quartz cuvette. ANS-binding assays (50 μM) were also performed on Triton X-100 (from 0 to 2 mM) and TAN (from 0 to 2 mM). Fluorescence intensities were corrected for volume changes and inner filter effects. Inner filter effect was corrected using the equation F_cor_ = F_obs_.10^(Aexc+Aem)/2^, where F_cor_, F_obs_, A_exc_ and A_em_ are fluorescence intensity corrected, fluorescence intensity observed, sums of the absorbances of enzyme and ANS at excitation wavelength and sums of the absorbances of enzyme and ANS at emission wavelength, respectively [31]. All the experiments were done in triplicate.

### Isothermal titration calorimetry (ITC)

Isothermal titration calorimetry (ITC) was carried out using a VP-ITC instrument from MicroCal (Northhampton, MA) at 25°C (298 K). To investigate the binding of tannic acid (TAN) to microbial β-glucosidases, the calorimetric cell (volume of 1.46 mL) was filled with 250 mM acetate-borate-phosphate buffer solution adjusted at different pH values (pH 6 for TpBGL1, pH 5 for PfGH1 and pH 4 for TpBGL3) containing 10 μM TpBGL1, TpBGL3 or PfBGL1. The buffer concentration used was chosen to avoid variations in the pH value with increasing TAN concentration. For the experiments with PfBGL1 and TpBGL3, the syringe was filled with 250 mM acetate-borate-phosphate buffer (adjusted at pH 5 for PfBGL1 and pH 4 for TpBGL3) containing 2 mM TAN and the titration was performed by 25 injections of 10 μL each into the sample cell. For the experiment with TpBGL1, the syringe was filled with 250 mM acetate-borate-phosphate buffer pH 6 containing 1 mM TAN, and the titration was performed by 50 injections of 5 μL each into the sample cell. A first 1 μL injection was performed in all titrations to minimize the volumetric error of the syringe plunger, and was later discarded in the analysis. In each case, the time between each injection was 600 s. The data obtained from isothermal titration were analyzed using the Origin software package for ITC analysis from Microcal (Microcal, Inc.). The data were fitted using the one-site model (identical and independent binding sites) [32]. The binding isotherms were analyzed by nonlinear regression to calculate the number of binding sites (*n*), the binding constant (*K*_*b*_), and the enthalpy of binding (Δ*H*). Thermodynamic parameters as changes in free energy (Δ*G*) and entropy (Δ*S*) of binding were determined from the Gibbs free energy relation Δ*G* = Δ*H*—TΔ*S* = -RTln(*K*_*b*_), where T is the absolute temperature and R = 1.987 cal/mol.K [33,34].

### Homology models and surface areas determination

We used the PISA server from the European Bioinformatics Institute [35] to determine the solvent-accessible surface area for each assembly. For PfBGL1 was used the PDB 3APG [36]. The three-dimensional models of the TpBGL1 and TpBGL3 were built by SWISS Model Server [37] using as template the available structures of the β-glucosidases from *Thermotoga maritima* (PDB 1OIN) [38] and *Thermotoga neapolitana* (PDB 2X41) [39], respectively.

## Results

### Inhibitory effects of tannic acid on microbial β-glucosidases

**Fig 1:**
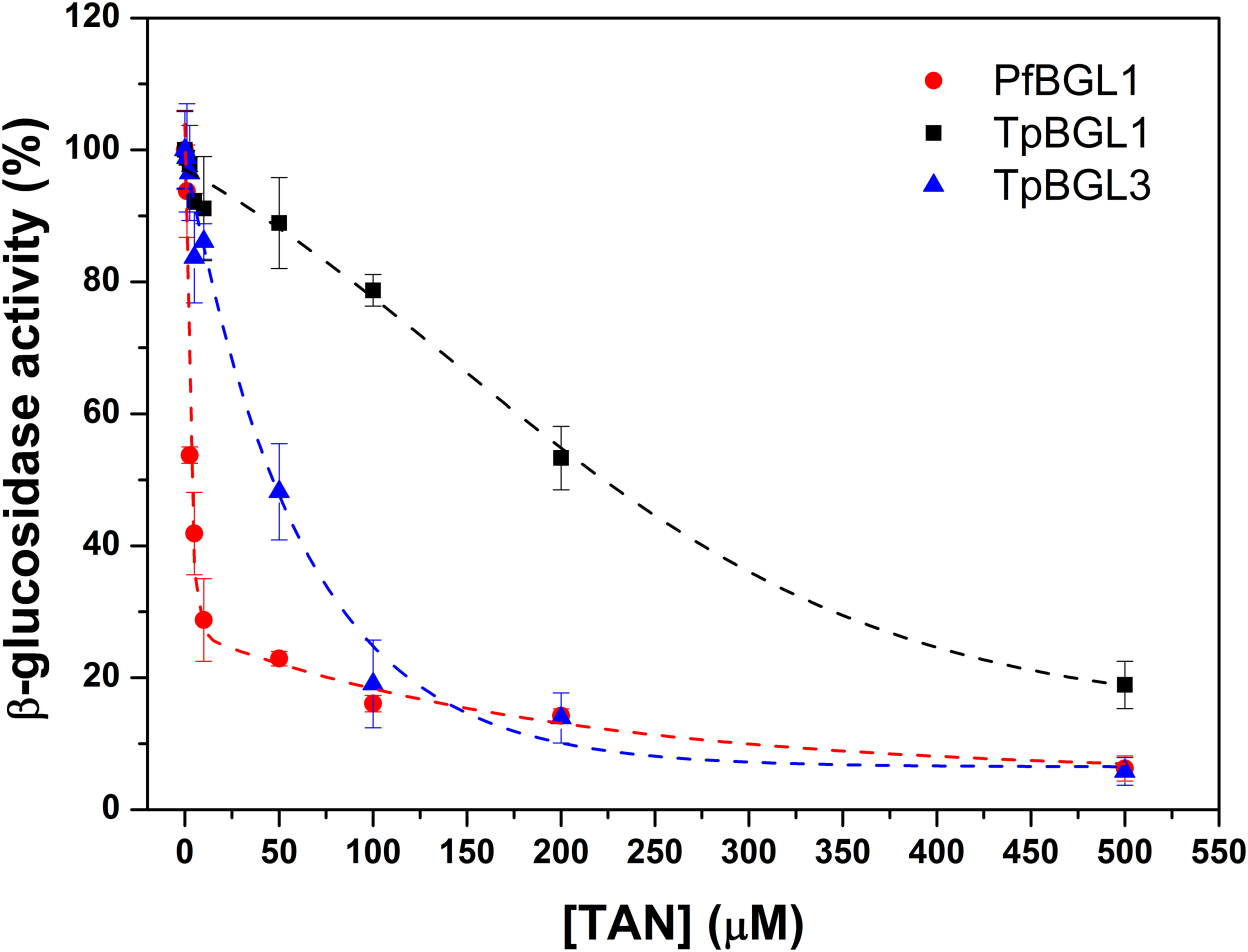
Inhibition assays. Effect of TAN concentration (from 0 to 0.5 mM) on the hydrolytic activities of the microbial β-glucosidases TpBGL1 (square), TpBGL3 (triangle) and PfBGL1 (circle). At fixed enzyme concentration of 1 μM was used.

The inhibitory effects of TAN on microbial β-glucosidases (enzyme concentration of 1 μM) were quantified by measuring the remaining hydrolytic activities after incubation the enzymes with different TAN concentrations (from 0 to 0.5 mM). As can be seen in Fig 1, the inhibition effects were found to be TAN concentration-dependent in all cases. Strong inhibitory effects on β-glucosidases were observed at the highest TAN concentration (0.5 mM) used in this study, with remaining activities lower than 20% in all cases. At TAN concentrations below 50 μM, both bacterial β-glucosidases (TpBGL1 and TpBGL3) were more tolerant to the presence of TAN when compared with archaeal β-glucosidase (PfBGL1). The remaining activities of TpBGL1, TpBGL3 and PfBGL1 were about 91%, 86% and 30% in the presence of 10 μM TAN, respectively. Furthermore, at TAN concentration of 100 μM bacterial β-glucosidase belonging to the family GH1 (TpBGL1) was less inhibited when compared with β-glucosidase belonging to the family GH3 (TpBGL3). The remaining activities of TpBGL1 and TpBGL3 were about 79% and 20% in the presence of 100 μM TAN, respectively (Fig 1).

### Influence of surfactant on microbial β-glucosidases

**Fig 2:**
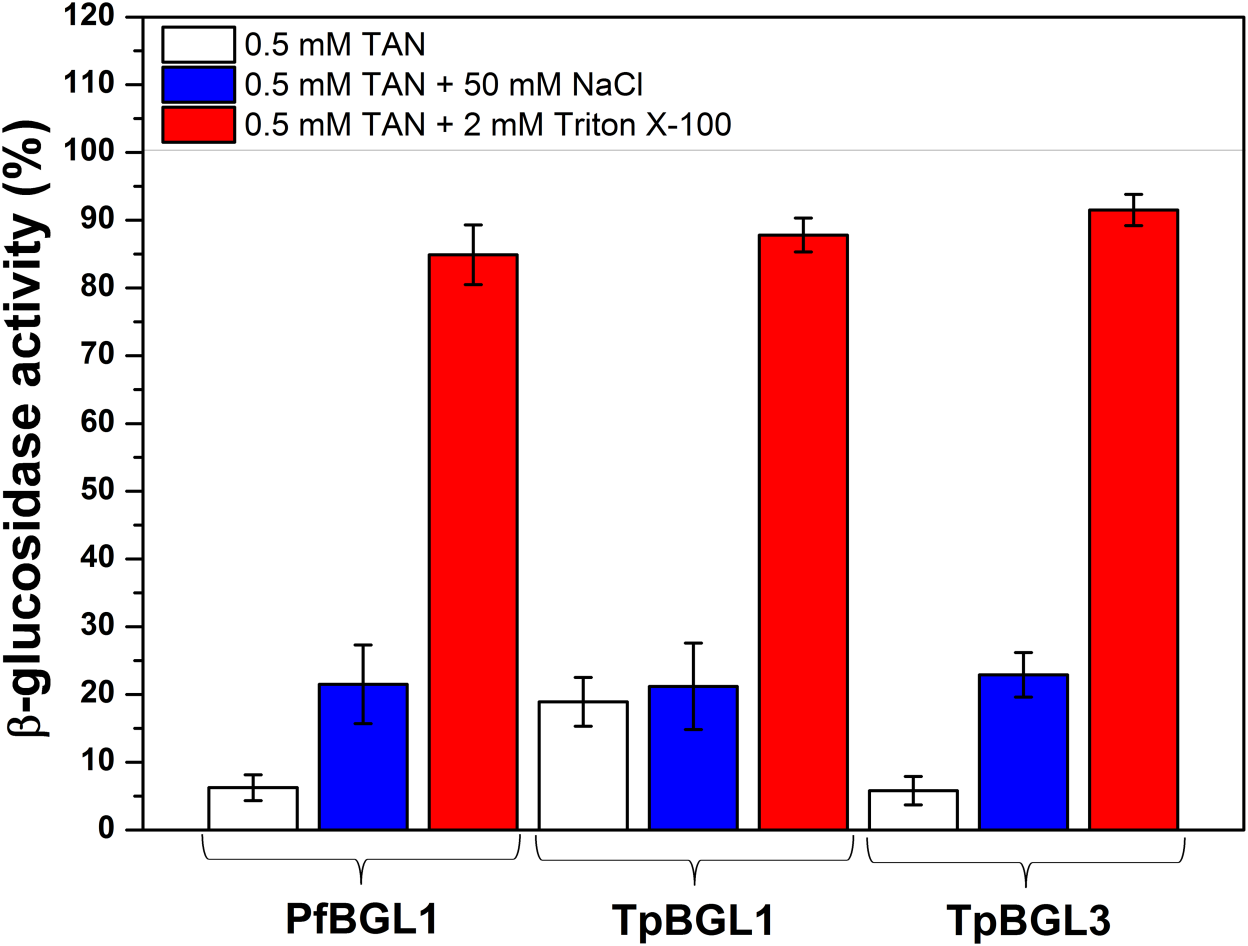
Influence of surfactant on β-glucosidases. Effect of Triton X-100 (2 mM) or NaCl (50 mM) on the hydrolytic activities of the β-glucosidases TpBGL1, TpBGL3 and PfBGL1 in the presence of 0.5 mM TAN. At fixed enzyme concentration of 1 μM was used.

As extensively reported in published studies, nonionic surfactants reduce or completely neutralize the inhibitory effect because the surfactant binds to TAN [18,19]. Thus, we measured the hydrolytic activities of microbial β-glucosidases in the presence of TAN in combination with Triton X-100 (Fig 2). As described above, in all cases, 0.5 mM TAN strongly reduced the β-glucosidases activities (Fig 1), while the hydrolytic activities were little altered (about 80–90%) by the addition of 2 mM Triton X-100. Furthermore, addition of 50 mM NaCl had little influence on the inhibitory effect of TAN (Fig 2). These results suggest that hydrophobic interactions contribute to the binding of TAN on microbial β-glucosidases.

### Exposed hydrophobic surface in microbial β-glucosidases

**Fig 3:**
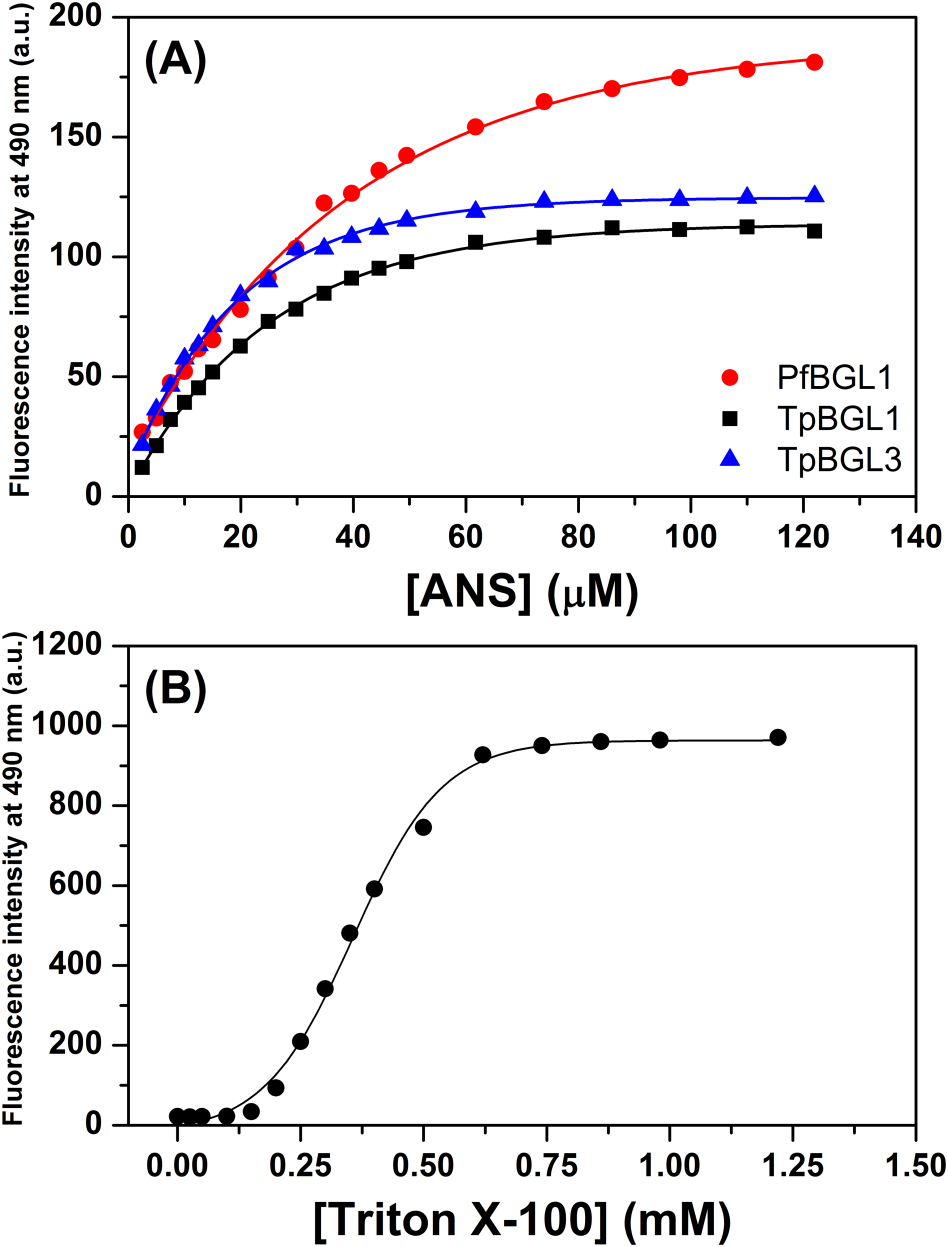
ANS assay. (**A**) Fluorescence emission representing ANS binding monitored at 490 nm using TpBGL1 (square), TpBGL3 (triangle) and PfBGL1 (circle). At fixed enzyme concentration of 10 μM was used for all β-glucosidases. ANS concentration was varied from 0 to 0.12 mM. (**B**) Fluorescence emission at 490 nm from ANS (50 μM) as a function of Triton X-100 concentration (from 0 to 1.25 mM).

In general, β-glucosidases have been described in various oligomeric states, including monomers, dimers, tetramers, hexamers, and octamers [40]. PfBGL1 and TpBGL1 are belonging to the family GH1 and have a common (α/β)_8_ barrel structure [26,36]. Belonging to the family GH3, TpBGL3 is arranged in three distinct domains: a N-terminal domain (α/β)_8_ triose phosphate isomerase barrel, followed by a five-stranded α/β sandwich, and a C-terminal fibronectin type III domain [26]. The archaeal PfBGL1 forms a very stable homotetramer in solution (S1 Fig) and the assembly has a solvent-accessible surface area of 64,660 Å^2^. The bacterial TpBGL1 and TpBGL3 are very stable homodimers in solution (S1 Fig) and have solvent-accessible surface areas of the assemblies of 29,990 and 50,060 Å^2^, respectively. To characterize the β-glucosidases in terms of exposed hydrophobic areas, we used the extrinsic fluorescent probe ANS. ANS binds noncovalently to hydrophobic surfaces, resulting in a pronounced increase in fluorescence emission when compared with the emission of free ANS in solution [29,30]. ANS-binding assays were performed on TpBGL1, TpBGL3 and PfBGL1 (Fig 3A). Excitation of the unbound ANS at 360 nm resulted in a very low fluorescent emission at 490 nm, however the fluorescent emission increased dramatically when the ANS bound to the hydrophobic regions of the β-glucosidases. The ANS fluorescence measurements for bacterial and archaeal β-glucosidases indicated more exposed hydrophobic surface in PfBGL1 when compared with TpBGL1 or TpBGL3. Furthermore, the results for bacterial β-glucosidases indicated more exposed hydrophobic surface in TpBGL3 when compared with TpBGL1.

### ANS interaction with nonionic surfactant and tannic acid

Results previously published reported that the addition of nonionic surfactant neutralizes completely the interaction between TAN and cellulases by a competition mechanism that favors the interaction between surfactant and TAN [18]. ANS-binding assays were performed on Triton X-100 and TAN to characterize the nature of these interactions. Thus, the ANS fluorescence emission was monitored in the absence and presence of Triton X-100 (or TAN). Excitation of the ANS (50 μM) in the presence of Triton X-100 at concentrations lower than 0.25 mM resulted in a low fluorescence intensity at 490 nm (Fig 3B). However, at concentrations higher than 0.25 mM the fluorescence emission at 490 nm increased progressively in intensity (Fig 3B), in agreement with the critical micelle concentration (CMC) for Triton X-100 [18]. These results confirmed that Triton X-100 can interact with ANS preferentially via hydrophobic interactions. Furthermore, results did not show a significant interaction between ANS and TAN (data not shown).

### Interactions between tannic acid and microbial β-glucosidases

**Fig 4:**
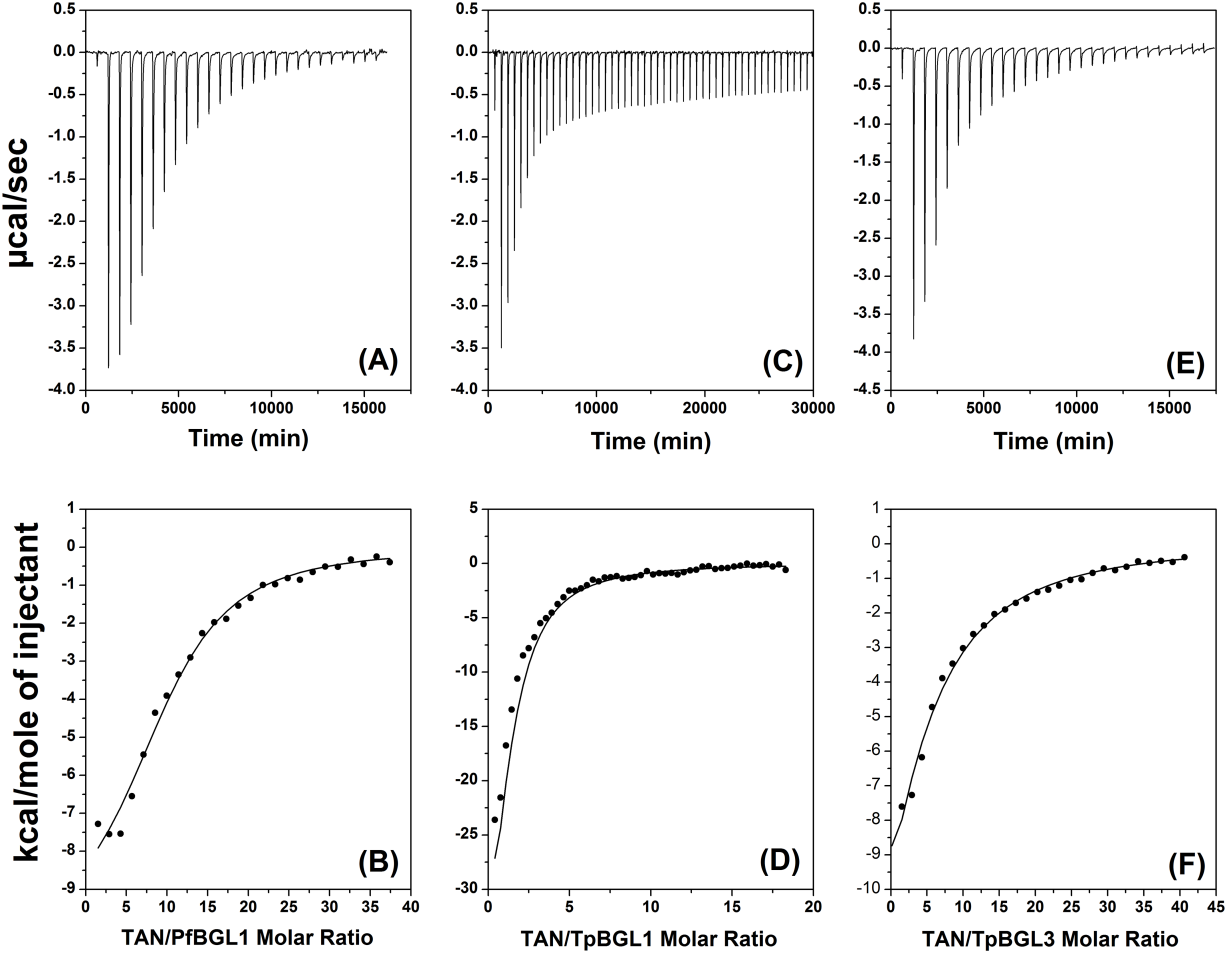
Isotherms for the calorimetric titrations of β-glucosidases with TAN at 25°C. (**A**) Raw calorimetric data of 25 x 10 μL injections of 2 mM TAN into 10 μM PfBGL1. (**B**) The heat of interaction as a function of the mole ratio of TAN/PfBGL1. (**C**) Raw calorimetric data of 50 x 5 μL injections of 1 mM TAN into 10 μM TpBGL1. (**D**) The heat of interaction as a function of the mole ratio of TAN/TpBGL1. (**E**) Raw calorimetric data of 25 x 10 μL injections of 2 mM TAN into 10 μM TpBGL3. (**F**) The heat of interaction as a function of the mole ratio of TAN/TpBGL3. The solid lines in (B), (D) and (F) are the best fits using the one-site model.

**Fig 5:**
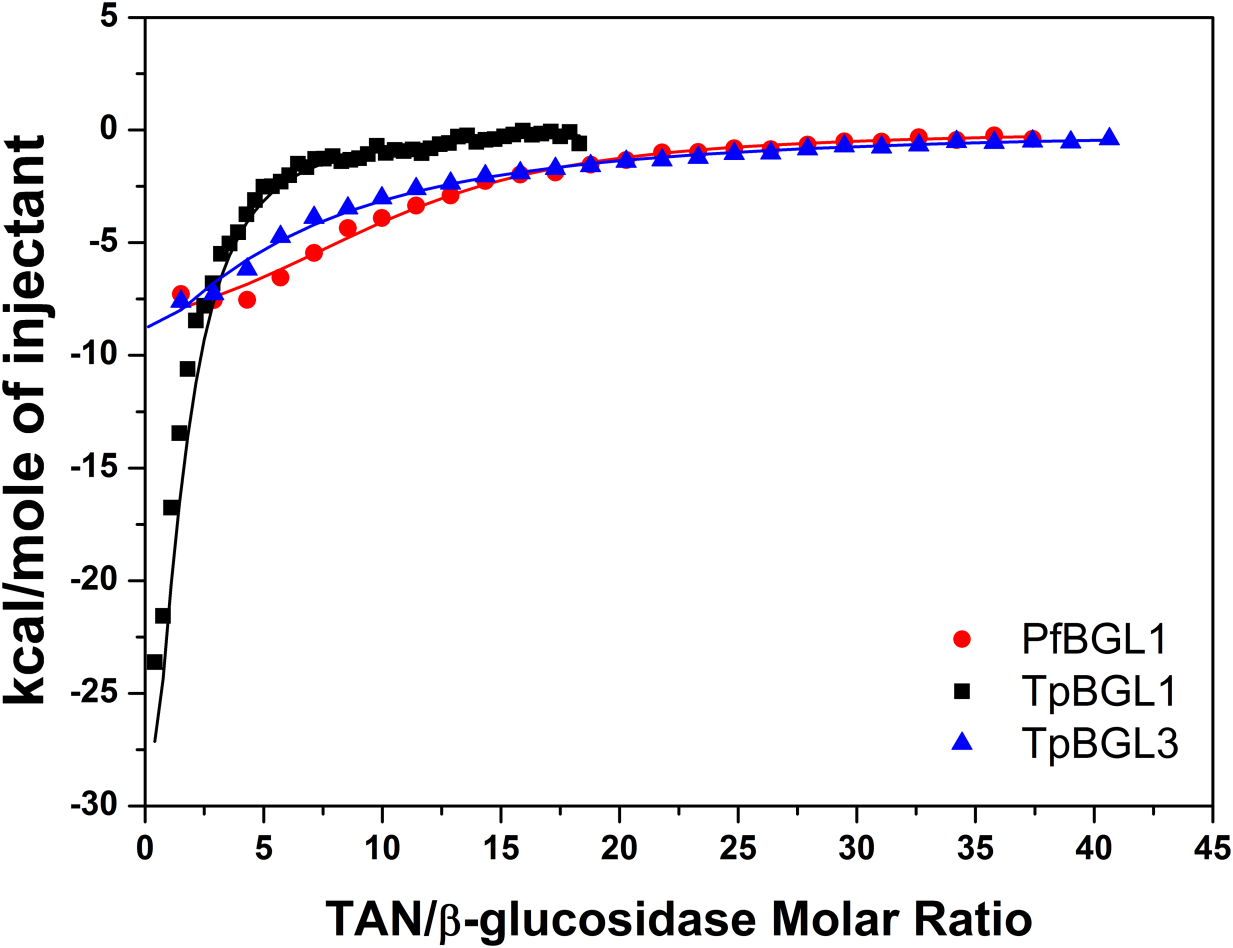
Calorimetric binding isotherms. The binding isotherms of TAN to PfBGL1 (sphere), TpBGL1 (square) and TpBGL3 (triangle). The solid lines are the best fits using the one-site model.

The interactions between TAN and microbial β-glucosidases were studied using ITC. Fig 4 shows the isothermal titration curves and the heat per injection obtained from the interaction between TAN and TpBGL1, TpBGL3 and PfBGL3. In all cases, each injection resulted in a negative deflection in the heat-flow consistent with exothermic interactions [18,32]. The TAN dilution heat was determined from the values of the last injections of each titration and was subtracted from the data shown in Fig 4 (bottom). For the determination of the parameters *n*, *K*_*b*_ and Δ*H*, the binding isotherms resulting from the interactions between TAN and microbial β-glucosidases were fitted using a model of identical and independent binding sites (one-site model) (Fig 5). All the thermodynamic parameters obtained from the fits are shown in Table 1. As can be seen from Table 1, the binding constants have the same order of magnitude (*K* ~ 10^4^ M^-1^) for all the β-glucosidases studied. The stoichiometries determined were about 1.5, 3.5 and 9.9 TAN bound per TpBGL1, TpBGL3 and PfBGL1, respectively. Furthermore, the values of Δ*G* and TΔ*S* were determined using the Gibbs free energy equation and are presented in Table 1.

**Table 1:** Summary of the thermodynamic parameters of binding of TAN to microbial β-glucosidases at 25°C. Binding constants calculated using ITC for the interaction between TAN and microbial β-glucosidases. *K*_*b*_ is the binding constant and *n* the binding stoichiometry. Δ*G*, Δ*H* and Δ*S* are the changes in the free energy, enthalpy and entropy of binding.

β-glucosidase	*K*_*b*_ (M^-1^ x 10^4^)	*n*	Δ*H* (kcal/mol)	Δ*G* (kcal/mol)	TΔ*S* (kcal/mol)
TpBGL1	7.4 ± 0.5	1.5 ± 0.5	-65.2 ± 7.1	-6.6 ± 0.1	-58.6 ± 0.7
TpBGL3	1.1 ± 0.2	3.5 ± 1.1	-32.2 ± 1.1	-5.5 ± 0.1	-26.7 ± 1.1
PfBGL1	3.6 ± 0.5	9.9 ± 0.5	-10.5 ± 0.6	-6.2 ± 0.1	-4.3 ± 0.6

## Discussion

This study aims to provide a comparative thermodynamic analysis of the interaction between TAN, a model polyphenolic compound, and β-glucosidases from different microorganisms and families. It is widely accepted that the presence of residual soluble phenols produced after pretreatment of lignocellulosic biomass can significantly reduce the enzymatic hydrolysis of cellulose to glucose [14–19]. Therefore, several studies have investigated the effect of polyphenolic compounds on enzymatic activity, and as a consequence, contributed to the improvement of cost-effective production of biofuels [14–19]. The archaeon *Pyrococcus furiosus* and the bacterium *Thermotoga petrophila* are hyperthermophilic microorganisms that produce various enzymes with potential industrial, including β-glucosidases [19,25,26,36,41,42].

The results showed that 0.5 mM of TAN was capable to inhibit almost completely the enzymatic activities of the microbial β-glucosidases used in this study (Fig 1). The ITC analysis showed that the binding between TAN and microbial β-glucosidases is exothermic (Table 1). The curves obtained for all β-glucosidases could be well fitted with a simple one-site model, indicating that all the binding sites are independent and thermodynamically equivalents. The binding constants determined from experimental data fits have the same order of magnitude (*K*_*b*_ ~ 10^4^ M^-1^), suggesting that most β-glucosidases in this study could form complexes with TAN at 0.5 mM (or higher concentrations). The binding constants determined here were an order of magnitude greater than observed for the interaction between TAN and β-glucosidase from *Aspergillus niger* (*K*_*b*_ = 1.8 x 10^3^ M^-1^) [18]. The complex formation may inhibit or deactivate the β-glucosidases to some extent. In comparison, TAN at a final concentration of 1.2 mM showed a strong inhibitory effect on β-glucosidase in a commercial preparation from *Aspergillus niger*, while the β-glucosidase from *Trichoderma reesei* was subtly inhibited [16]. Furthermore, in the presence of 0.25 mM TAN a strong inhibitory effect was reported on β-glucosidase 1 from *Saccharomycosis fibuligera*, while at a concentration of 1 mM the remaining activity was virtually zero [43]. Also, a tannase from *Aspergillus niger* with β-glucosidase activity (the enzyme was capable to hydrolyze cellobiose into glucose) was completely inhibited in the presence of 50 mM TAN [44]. Residual soluble phenolics derived from lignin after industrial pretreatment are present in concentrations between 10^−6^ and 10^−3^ M, concentration range that may affect the hydrolytic activity due to the formation of complexes between TAN and β-glucosidases [15,16]. For the β-glucosidases analyzed in this study, the addition of Triton X-100 neutralized almost completely the inhibitory effect of TAN (Fig 2) suggesting that hydrophobic interactions contribute to the binding of TAN on microbial β-glucosidases, in agreement with published studies [18,19].

**Fig 6:**
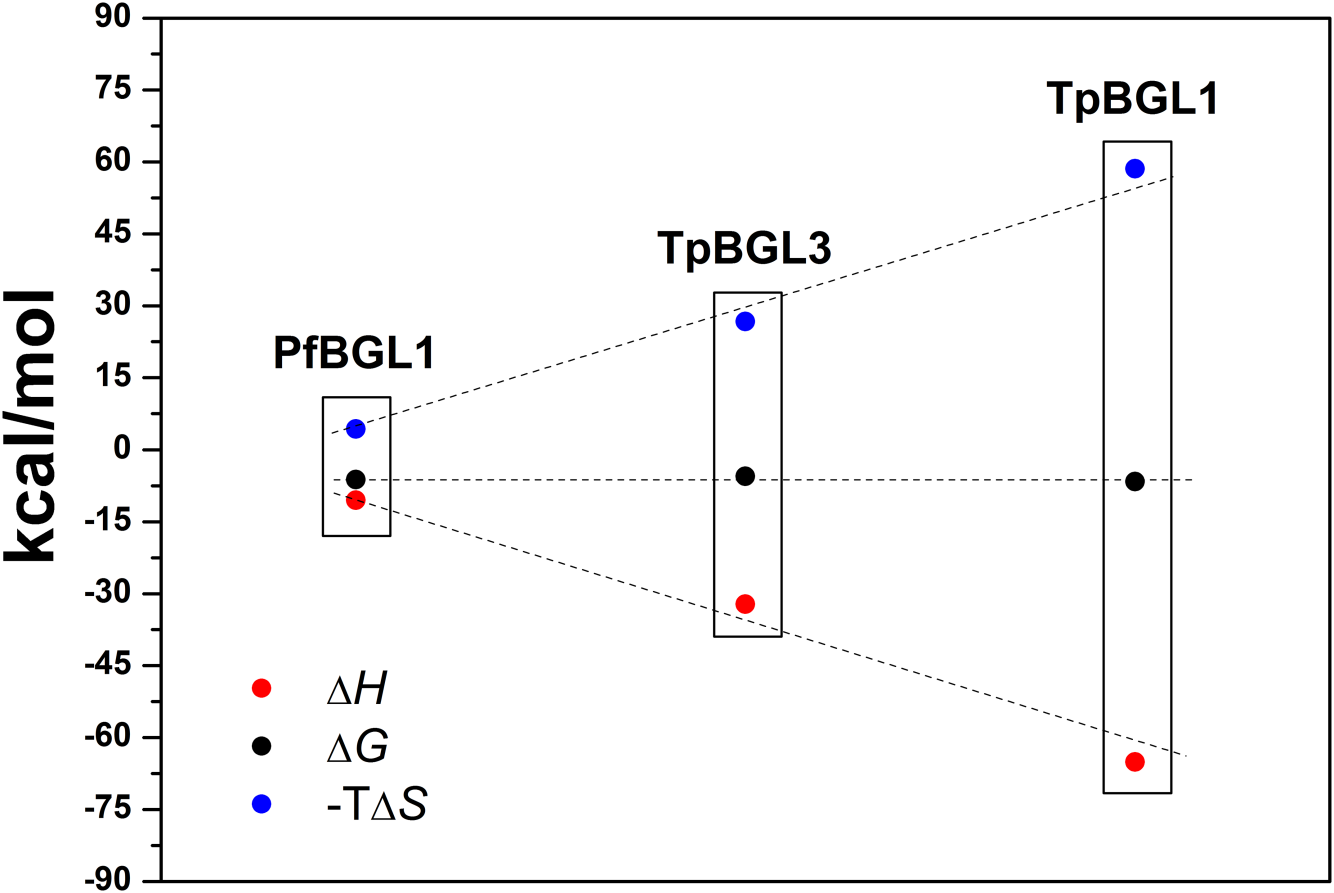
Free energy. The changes in the free energy (Δ*G*), enthalpy (Δ*H*) and entropy (Δ*S*) for binding of TAN to PfBGL1, TpBGL1 and TpBGL3.

According to our data, the binding of TAN to all β-glucosidases is associated with favorable enthalpy change (negative Δ*H*) and unfavorable entropy change (negative Δ*S*). TpBGL1 had the highest negative binding enthalpy followed by TpBGL3 and PfBGL1, respectively (Table 1). Since the binding constants obtained for the three β-glucosidases were basically the same, Δ*G* values remained practically invariant. Therefore, the Δ*H* values were almost canceled by the associated unfavorable TΔ*S* (Fig 6). The negative Δ*H* values are associated with van der Waals interactions and hydrogen bonding, while negative Δ*S* values are associated with the net formation of hydrogen bonds, and decrease in the number of conformations and degrees of freedom [33]. It is reasonable to consider that the binding enthalpy between TAN and β-glucosidases occurs with the formation of hydrogen bonds (polar interactions) and with increased hydrophobic interactions, therefore driven by enthalpic effects and with an unfavorable negative change in entropy upon binding [33,34,45]. The hydrophobic interactions may be related with the release of structured water molecules from hydrophobic surfaces of TAN and β-glucosidases during the formation of the complexes [33,34,45]. ANS-binding assays showed that all the β-glucosidases in this study exhibited hydrophobic surfaces on the native structures exposed to the solvent (Fig 3A). Similarly, it was reported that polyphenols bind to casein and β-lactoglobulin through both hydrophilic and hydrophobic interactions [24].

Interestingly, at TAN concentrations below 0.5 mM the inhibitory effects were found to be concentration-dependent (Fig 1). The results showed that both bacterial β-glucosidases were more tolerant to the presence of TAN when compared with archaeal β-glucosidase. Furthermore, *T*. *petrophila* β-glucosidase belonging to the family GH1 was less inhibited when compared with β-glucosidase belonging to the family GH3. In addition, the number of binding sites (Table 1) and exposed hydrophobic surfaces areas (Fig 3A) varied for the β-glucosidases under study. The interaction between polyphenols and proteins is affected by the types of protein folds, oligomerization states and the molar ratio of phenolic/protein [24]. Also, it is known that polyphenols to form complexes with proteins (increasing the molecular mass of proteins) which may lead to changes in the structural and functional properties [24]. For example, casein and β-lactoglobulin conformations were changed by polyphenols with alterations in the content of regular secondary structures [24]. The PfBGL1 showed greater exposed hydrophobic surface area, number of binding sites and inhibition by TAN when compared with bacterial β-glucosidases. The stoichiometry was 9.9 TAN bound per PfBGL1, leading to an increase in the molecular mass of 31%. PfBGL1 forms a homotetramer in solution, therefore each PfBGL1 oligomer bind approximately 40 molecules of TAN indicating that the oligomer surface was covered by polyphenols. Complex formation of β-glucosidases and TAN may modify the net charge and the structural conformation of the enzyme and directly affect its hydrolytic activity. For bacterial β-glucosidases, TpBGL1 showed lower exposed hydrophobic surface area, number of binding sites and inhibition by TAN when compared with TpBGL3. The stoichiometries were about 1.5 and 3.5 TAN bound per TpBGL1 and TpBGL3, leading to an increase in the molecular mass of only 5% and 7%, respectively. The data provided here suggest that there is a high correlation between exposed hydrophobic surface areas and the number of binding sites on the inhibition of microbial β-glucosidases by TAN. A larger number of exposed hydrophobic surface areas are susceptible to binding of a higher number of TAN molecules and consequently to a greater inhibition of the hydrolytic activity.

## Conclusions

In conclusion, the inhibitions of microbial β-glucosidases were found to be TAN concentration-dependent. The bacterial β-glucosidases were less inhibited by TAN when compared with archaeal β-glucosidase, while bacterial TpBGL1 was less inhibited when compared with TpBGL3. The thermodynamic analyzes showed that the binding of TAN to the β-glucosidases was driven by enthalpic effects with an unfavorable negative change in entropy upon binding. The binding between TAN and β-glucosidases occurs with the formation of hydrogen bonds and increased hydrophobic interactions. Taken together, our studies of the interaction between TAN and microbial β-glucosidases suggest that there is a high correlation between exposed hydrophobic surface areas and the number of binding sites on the inhibition of β-glucosidases by TAN. These data may provide a useful basis for future biotechnological applications of microbial β-glucosidases, especially in the field of biofuel production.

## Supporting information

S1 Fig Oligomeric states and molecular surface representation of β-glucosidases. (**A**) PfBGL1 (homotetramer, PDB 3APG) [36]. (**B**) TpBGL1 (homodimer). (C) TpBGL3 (homodimer). Hydrophobic residues are highlighted in orange. The homology models of the TpBGL1 and TpBGL3 were built using as template the available structures of the β-glucosidases from *Thermotoga maritima* (PDB 1OIN, 99% sequential identity) [38] and *Thermotoga neapolitana* (PDB 2X41, 88% sequential identity) [39], respectively.
(TIF)
